# Propranolol for the Treatment of Hemangioma in PHACE Syndrome: A Case Report

**DOI:** 10.7759/cureus.44036

**Published:** 2023-08-24

**Authors:** Tala Beidas, Yara Jazzar, Asem Shadid, Abdulmalik Alhammad, Khaled A Mohajer, Amr M. Abduljabbar

**Affiliations:** 1 Dermatology, King Saud Medical City, Riyadh, SAU; 2 Dermatology, Alfaisal University College of Medicine, Riyadh, SAU; 3 Dermatology, King Fahad Medical City, Riyadh, SAU

**Keywords:** cleft lip, dandy-walker, propranolol, phace syndrome, hemangioma

## Abstract

Oral propranolol is commonly used as a first-line treatment for infantile hemangioma. However, its use in PHACE (posterior fossa anomalies, hemangioma, arterial anomalies, cardiac anomalies, and eye anomalies) syndrome raises concerns that it might exacerbate the patient’s risk of stroke. Here, we report the case of a four-month-old premature girl with PHACE syndrome, who presented with a large hemangioma involving the left side of her face, following the V1+V2+V3 distribution, including the upper lip, left ear, and left eye. This condition was successfully treated with propranolol, and no adverse side effects were reported.

## Introduction

PHACE (posterior fossa anomalies, hemangioma, arterial anomalies, cardiac anomalies, and eye anomalies) syndrome is a rare disorder characterized by large segmental hemangiomas on the face and is associated with multiple developmental defects. Since its initial description, significant advances have been made in understanding the condition [1]. Oral propranolol is a commonly used first-line treatment for infantile hemangioma (IH). However, its use in PHACE syndrome has been the subject of intense debate due to concerns that β-blockers might increase the risk of stroke in these patients. This is especially challenging because most PHACE syndrome patients present with extensive facial IH and multiple morbidities [1,2]. Nevertheless, aggressive treatment is necessary to address the rapid proliferation of IHs in PHACE syndrome patients, leading to increased clinical use of oral propranolol in recent years. In this case, we present a 4-month-old girl diagnosed with PHACE syndrome, who had a large hemangioma involving the left side of her face following the (V1+V2+V3) distribution, including the upper lip, left ear, and left eye. The patient was successfully treated with propranolol, and no adverse effects were reported.

## Case presentation

**Figure 1:**
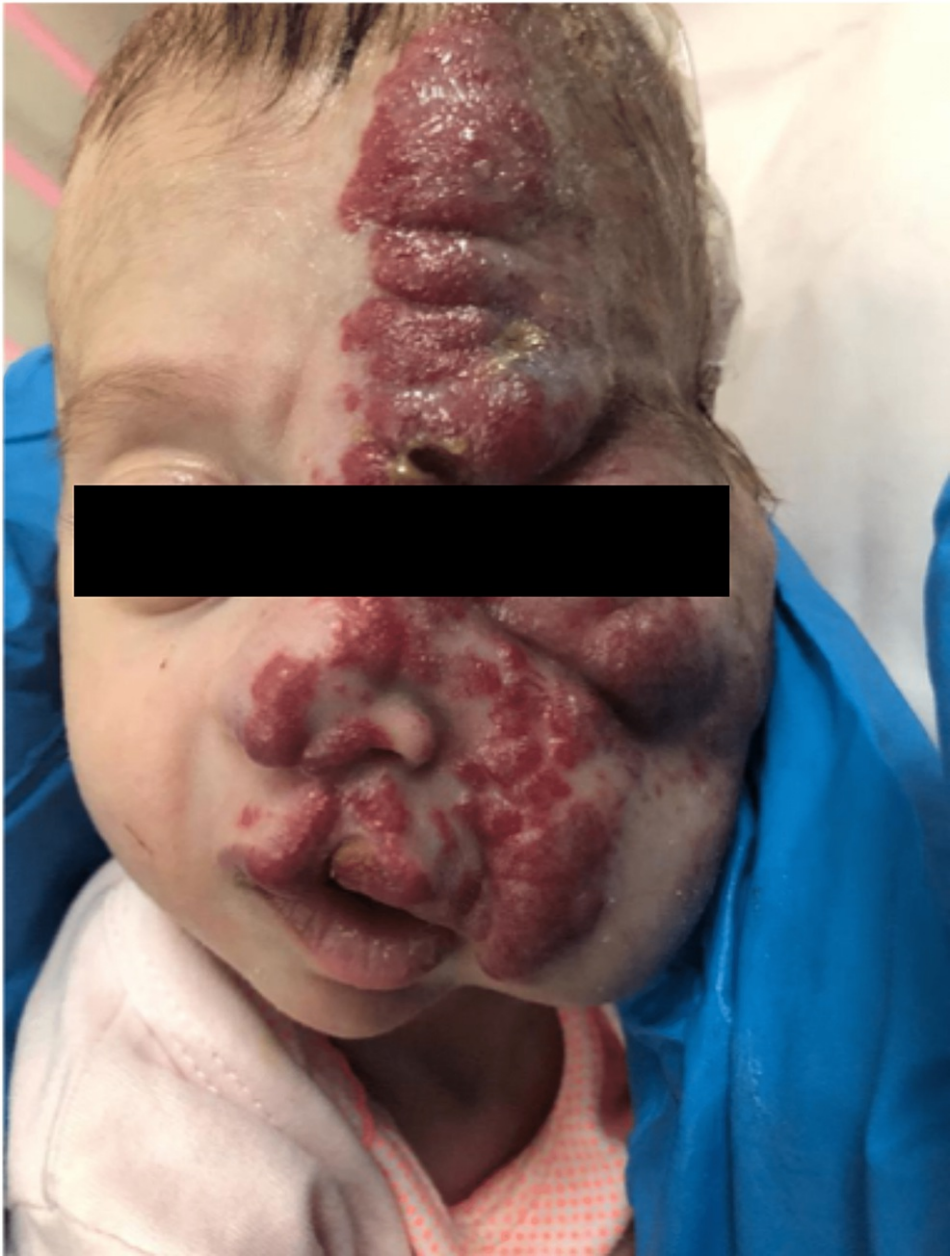
At four months, before starting treatment, with pus discharge from the lesion.

The female child was born via normal vaginal delivery at 32 weeks of gestation following a complicated pregnancy. At 29 weeks, the baby was in the vertex position, and the cervix was noted to be incompetent. Three weeks before delivery, she received a steroid to promote lung maturity. She was the second child of a 19-year-old gravida 2, para 2 mother and a 30-year-old father. The parents are first-degree cousins, and there were no remarkable family history issues. The baby’s birth weight was 1.9 kg, and she was admitted to the Neonatal Intensive Care Unit for 40 days due to hemifacial pigmentation. At 41 days old, she was referred to our hospital for further evaluation as her main hospital lacked neurosurgeons, and she had a noticeable progressive increase in head size when she was 30 days old (in the 97th percentile head circumference). During our initial examination, at 41 days old, all her vitals were within normal range, and her head circumference measured 40 cm (in the 97th percentile head circumference). We observed a wide (3 x 4 cm) bulging, tense anterior fontanelle and noted mild hypotonia in her lower limbs. On chest inspection, minimal pectus excavatum had normal s1+s2 sounds and no additional abnormalities. We observed a well-defined bright red infiltrating plaque over the left side of her face following the V1+V2+V3 distribution, including the upper lip (cleft lip), left ear, and left eye (Figure 1).

**Figure 2:**
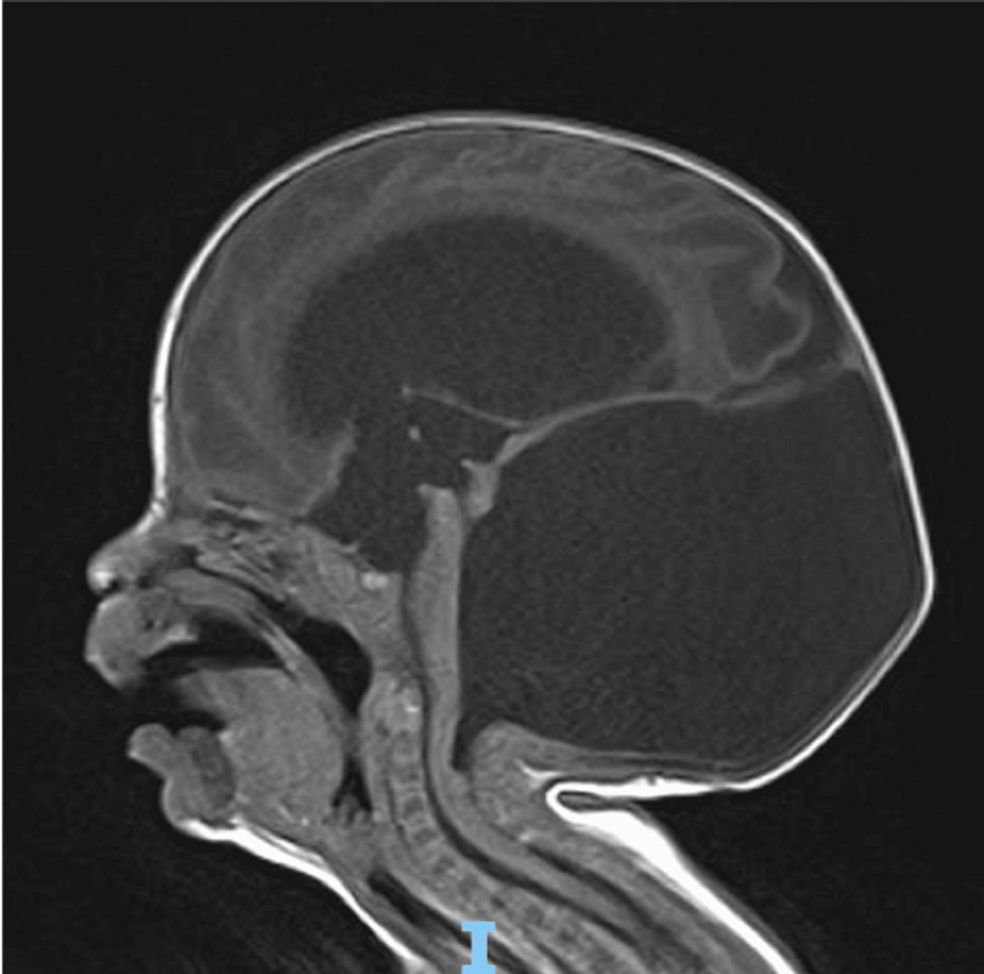
MRI of the brain showing marked hydrocephalus with a severely dilated fourth ventricle occupying the enlarged posterior fossa.

She was born with a dusky red discoloration over the left half of her face, but this lesion has been progressively growing and enlarging over time. Additionally, there was a yellowish discharge from the lesion, and a swab revealed the presence of *Staphylococcus aureus*. Her genitourinary examination noted edematous labia majora. She was admitted with a diagnosis of Dandy-Walker malformation and hydrocephalus, which required a ventricular tap. Upon admission, a computerized tomography (CT), magnetic resonance imaging (MRI), and echocardiogram (Echo) were ordered. The non-contrast brain CT revealed severe communicating hydrocephalus with cystic dilation of the posterior fossa. The multisequence multiplanar brain MRI with and without IV gadolinium showed marked hydrocephalus in the supratentorial ventricular system, along with a severely dilated fourth ventricle occupying the enlarged posterior fossa and a hypoplastic cerebellum. A suspicious syrinx was visualized in the cervical cord, and bilateral check lobulated soft tissue lesions extending to the pterygoid fossa, larger on the left side, were observed, likely representing soft tissue hemangiomas. The periventricular hypodense white matter could be related to the patient’s premature age or periventricular interstitial edema (Figure 2). No evidence of midline shift, intracranial hemorrhage, or restriction was detected. The Echo showed good cardiac function, a patent foramen ovale with a left-to-right shunt, and a normal aortic arch, with the rest of the study being normal.

**Figure 3:**
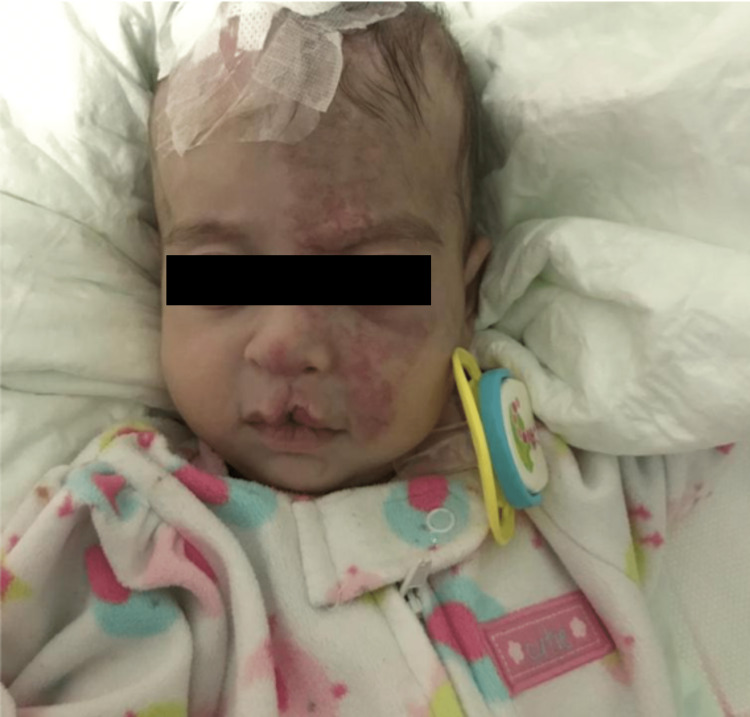
A few months after starting treatment.

**Figure 4:**
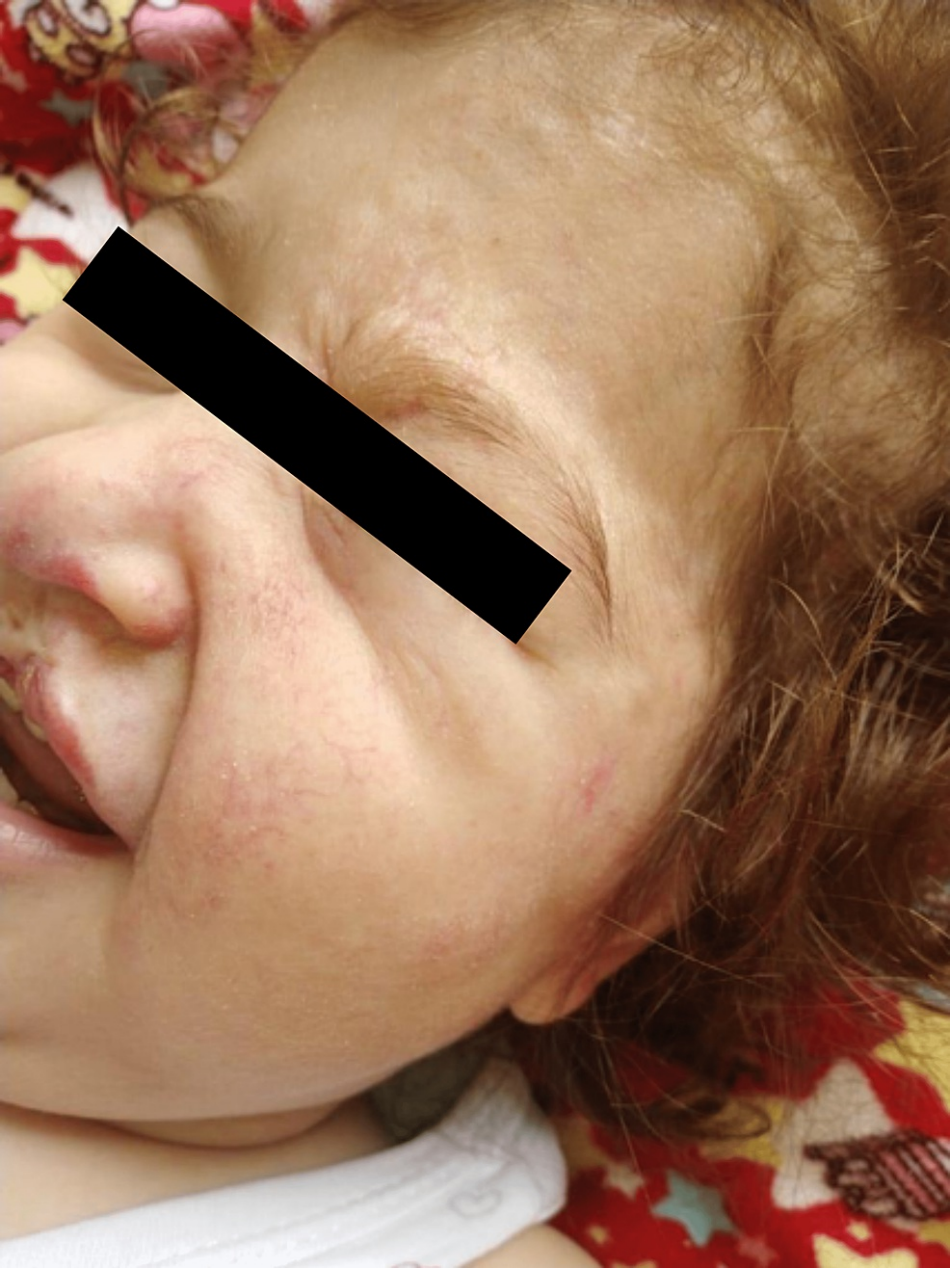
After two years of treatment, IH was cleared

The day after her admission, an urgent external ventricular drain (EVD) was inserted by the Neurosurgery team. However, the EVD was complicated by leaking and fever after only a few days, necessitating a replacement. Ophthalmology consultation revealed no eye anomalies except the hemangioma involving the left eye, which caused proptosis, but later imaging confirmed its clearance. Subsequently, the EVD was removed, and a ventriculoperitoneal (VP) shunt was inserted three months after the patient’s stabilization and discharge. Unfortunately, the VP shunt experienced blockage and required replacement shortly after the patient was discharged. She then began following up with dermatology as an outpatient. Once the patient was cleared by cardiology, we initiated propranolol treatment at a dose of 0.75 mg/kg/day, measuring her heart rate and blood pressure as baseline parameters, two hours after the initial dose and with each visit. Administered three times a day (TID). Gradually, the dosage was increased to 3 mg/kg/day, TID. Over the course of 22 months, the patient’s hemangioma completely cleared (Figures 3, 4).

## Discussion

PHACE syndrome, specifically IH, was initially treated with oral corticosteroids. However, their low efficacy and high risk of adverse effects, including an increased risk of stroke, made them less than ideal as a first-line treatment. This prompted some clinicians to consider using propranolol alone or in combination with oral corticosteroids when the latter failed to effectively treat the patient [3,4]. The introduction of propranolol for IH in 2008 marked a significant turning point in IH management, leading to dramatic improvements in outcomes for most infants who tolerated the treatment well [5].

However, PHACE syndrome patients presented a challenging situation as they often had extensive facial IH with multiple morbidities, making them prime candidates for propranolol therapy while simultaneously raising concerns about potential devastating complications. Though the incidence of stroke in the pediatric population is rare, the presence of cerebrovascular abnormalities places these patients at a higher risk, as it is recognized in the [Figure 4].

After 2 years of treatment, IH was cleared, with the most critical risk factor for stroke being structural cerebral and cerebrovascular arterial anomalies that are common features of PHACE syndrome [5,6,7], further elevating the risk of stroke in affected individuals. Initially, there was reluctance to use propranolol in PHACE syndrome, mainly due to theoretical concerns that β-blockers could decrease cardiac output, reducing perfusion in the cerebral arteries and causing infarction in areas with absent, stenosed, or occluded arteries [1].

Fortunately, the hemodynamic changes in infantile blood pressure and perfusion are more complex, as demonstrated by several studies that indicated the clinical insignificance of propranolol on these factors. A 2019 cohort study of 76 PHACE patients on oral propranolol therapy reported no incidence of stroke but highlighted the need for careful physical examination and echocardiography to rule out aortic arch coarctation. Additionally, large blood pressure fluctuations were prevented by initiating the treatment at the lowest possible dose and administering it three times daily [1,6].

Several cases have shown remarkable improvement and good tolerance with propranolol treatment [3,6]. Multiple studies have also reported the possibility of IH recurrence and tumor growth upon discontinuation of propranolol treatment, particularly if the treatment was halted prematurely [6]. Therefore, the duration of propranolol treatment should be based on the clinical progression of the IH, and treatment should be discontinued when no further reduction in the lesion is observed while on medication. Follow-up of the patient after treatment discontinuation is crucial, as re-initiating treatment may be necessary in cases of recurrence [6].

## Conclusions

We present the case of a four-month-old girl diagnosed with PHACE syndrome, who had a sizable hemangioma affecting the left side of her face, following the V1+V2+V3 distribution, including the upper lip, left ear, and left eye. We initiated her treatment cautiously, starting with a dosage of 0.75 mg/kg/day of propranolol administered three times daily, gradually increasing to 3 mg/kg/day, closely monitoring her progress throughout. Over the course of two years, the patient’s hemangioma showed remarkable improvement with no reported adverse effects.

## References

[1] Olsen GM, Hansen LM, Stefanko NS (2020). Evaluating the safety of oral propranolol therapy in patients with PHACE syndrome. JAMA Dermatol.

[2] Metry D, Frieden IJ, Hess C (2013). Propranolol use in PHACE syndrome with cervical and intracranial arterial anomalies: collective experience in 32 infants. Pediatr Dermatol.

[3] Bellaud G, Puzenat E, Billon-Grand NC, Humbert P, Aubin F (2015). PHACE syndrome, a series of six patients: clinical and morphological manifestations, propranolol efficacy, and safety. Int J Dermatol.

[4] Gnarra M, Solman L, Harper J, Batul Syed S (2015). Propranolol and prednisolone combination for the treatment of segmental haemangioma in PHACES syndrome. Br J Dermatol.

[5] Siegel DH (2023). PHACE syndrome. UpToDate.

[6] Rotter A, Samorano LP, Rivitti-Machado MC, Oliveira ZN, Gontijo B (2018). PHACE syndrome: clinical manifestations, diagnostic criteria, and management. An Bras Dermatol.

[7] Bhattacharya JJ, Luo CB, Alvarez H, Rodesch G, Pongpech S, Lasjaunias PL (2004). PHACES syndrome: a review of eight previously unreported cases with late arterial occlusions. Neuroradiology.

